# Nonverbal Response Cards Reduce Socially Desirable Reporting of Violence Among Adolescents in Rural Burkina Faso: A Randomized Controlled Trial

**DOI:** 10.1016/j.jadohealth.2020.09.006

**Published:** 2021-05

**Authors:** Guy Harling, Mamadou Bountogo, Ali Sié, Till Bärnighausen, David P. Lindstrom

**Affiliations:** a Institute for Global Health, University College London, London, United Kingdom; b Africa Health Research Institute (AHRI), KwaZulu-Natal, South Africa; c Department of Epidemiology, Harvard Center for Population and Development Studies, Harvard T.H. Chan School of Public Health, Boston, Massachusetts; d MRC/WITS Rural Public Health and Health Transitions Research Unit (Agincourt), Faculty of Health Sciences, University of the Witwatersrand, Johannesburg, South Africa; e Centre de Recherche en Santé de Nouna, Nouna, Burkina Faso; f Heidelberg Institute of Global Health (HIGH), University of Heidelberg, Heidelberg, Germany; g Department of Global Health and Population, Harvard T.H. Chan School of Public Health, Boston, Massachusetts; h Department of Sociology, Population Studies and Training Center, Brown University, Providence, Rhode Island

**Keywords:** Adolescent health, Social desirability bias, Physical violence, Sexual violence

## Abstract

**Purpose:**

Accurate measures of violence are difficult to obtain from self-reported data because of stigmatization and social undesirability of the topic. Most methods that attempt to reduce such biases require literacy and either remove the benefits of interviewer guidance or do not give individual-level results. We tested a low-tech nonverbal response card that avoids revealing interviewees' responses to interviewers while retaining interviewer support among adolescents in communities with very low educational attainment.

**Methods:**

As part of a broader health questionnaire, we asked a sample of 1,644 adolescents, aged 12–20 years, in northwestern Burkina Faso about their experiences of physical and sexual violence. We randomized participants to either a conventional verbal response arm or a nonverbal response card arm where respondents' answers were unspoken and not displayed to interviewers. We first evaluated response validity and reliability in each arm, then compared prevalence rates across arms and evaluated whether any differences varied by respondent characteristics using regression models.

**Results:**

The level of internal reliability of responses among nonverbal respondents was similar to or greater than that of verbal respondents. Nonverbal respondents reported similar patterns of physical assault and sexual debut as verbal respondents but significantly higher levels of sexual assault and forced sex. These differences were broadly similar across sample subgroups defined by age, gender, proneness to social desirability, and mental health.

**Conclusions:**

Nonverbal response cards offer a practical and beneficial method for reducing underreporting of stigmatized and traumatic experiences while maintaining data quality in low-literacy populations.

**Implications and Contribution**

This study shows that the nonverbal response card method for interviewer-administered survey questionnaires provides valid and likely more accurate estimates of sexual violence prevalence than conventional verbal responses. The nonverbal response method can help improve understanding of stigmatized, socially undesirable experiences and behaviors in low-literacy populations by providing more accurate estimates.

Violence, enacted either by intimate partners or others, is both highly prevalent and has substantial effects on the lives of survivors. The World Health Organization estimates that 35% of women have experienced either physical violence from an intimate partner or sexual violence; estimates for West Africa are above the global average for both intimate partner and nonpartner sexual violence [1]. Although fewer men are survivors of sexual violence, many still experience being forced to penetrate someone else, being sexually coerced, or facing unwanted sexual contact or noncontact sexual experiences [2]. Self-reported history of forced sex among young men in Africa ranges up to 30% [3,4], with almost 5% of young Burkinabe men reporting being “not willing at all” at sexual debut [5]. Such violence has myriad physical and psychological impacts [6,7]. Violence against children can be particularly debilitating insofar, as it affects them during a key developmental period, affecting health, learning, and future life opportunities [8]. A meta-analysis of population-based studies estimated that by age 18 years, 18.0% of girls and 7.6% of boys had experienced sexual abuse alone [9].

The prevalence of violence and associated risk factors is difficult to estimate reliably because it is almost always based on retrospective self-reports and thus susceptible to both inadvertent recall bias and intentional “social desirability” bias, that is, the tendency to underreport stigmatized (and overreport socially normative) behaviors. Intentional underreporting may be exacerbated by the highly sensitive and stigmatizing nature of sexual violence and the degree to which violence is inflicted by family members and other loved individuals [10]. As a result, the true prevalence of violence and risk factors predicting it remains somewhat uncertain.

Social desirability bias is well known in the sexual behavior and survey literature [11,12]. Several methods have been proposed for reducing the risk of intentional misreporting in surveys. One such approach is to ask respondents to record their answers either on paper or on a computer screen, that is, complete a “self-interview” or to post their answer in a ballot box [13]. These methods require literacy and potentially experience and comfort with computers, which is not universal in many low-income settings. Another approach is to hide the respondents' answer to the sensitive question among other answers. One hidden response approach is the “random response” technique, where respondents answer truthfully some known proportion of the time, but the interviewer does not know when [14]. For example, before answering, the respondent rolls a die; if it shows a “1,” they always say “no”; if it shows “6,” they always say “yes,” otherwise they tell the truth. Another hidden response method is the “list randomization” technique, where the respondent is asked how many items from a list of statements they agree with [15]; respondents are randomized to either have or not have a sensitive question added to their list. Both methods allow for calculation of the proportion of people affirming the sensitive question. These hidden response methods, while potentially effective at estimating population prevalence, do not allow post-hoc identification of individual's specific responses for use in the analysis.

The nonverbal response card (NVRC) is a method for reducing social desirability bias that can be used in low-literacy settings and allows individual responses to be recovered. These cards were developed for soliciting responses to questions about sexual knowledge, attitudes, and practices among adolescents in Ethiopia [16,17]. The NVRC method allows respondents to indicate their response to interviewers, without the interviewers knowing their specific answer. The cards are low tech, lightweight, privacy preserving, and require minimal literacy and thus appropriate for fieldwork in low-literacy populations. Previous trials comparing the cards to conventional verbal responses found significantly higher reported prevalence of sexual coercion, rape, and nonmarital sex and lower reported levels of condom knowledge among respondents who used the card method [16,17].

The NVRC method has not, however, been tested outside of Ethiopia. We conducted an individually randomized experiment of using NVRCs to ask adolescents in rural and semirural Burkina Faso about behaviors that are likely stigmatized and socially undesirable to report on physical and sexual violence.

## Methods

### Setting and sample

Our data comprise baseline responses from a cohort of young people aged 12–20 years in rural Burkina Faso. The cohort forms part of the Africa Research, Implementation Science, and Education (ARISE) network, a collaboration between nine sub-Saharan African institutions in seven countries, Harvard T.H. Chan School of Public Health, and the University of Heidelberg [18]. The Burkinabe cohort was drawn from the population of the Health and Demographic Surveillance System (HDSS) run by the Centre de Recherche en Santé de Nouna (CRSN). We have previously used data from this cohort to identify risk factors for adolescent health [19,20]. The HDSS community (total population approximately 107,000 in 2015) is centered on the town of Nouna in the Boucle du Mouhoun province in northwestern Burkina Faso and comprises the town of Nouna (population approximately 30,000) and 58 surrounding rural villages; a mixture of ethnic and religious groups live in the community [21].

ARISE Burkina Faso used a two-part stratified sampling procedure to ensure the representativeness of ethnicity/religion and urbanicity. We first purposively selected 10 Nouna HDSS villages such that each of the five main ethnicities formed a majority in two. We then drew a random sample of 1,795 youth from a 2015 census of residents of these 10 villages who were age eligible on October 1, 2017, ensuring this sample respected the ethnic distribution of all age-eligible HDSS adolescents. Second, we drew a simple random sample of 749 age-eligible adolescents from one of the seven sectors of Nouna town.

Baseline face-to-face interviews were conducted in November and December 2017 in the compounds where the adolescents lived or at another location chosen by the participant. Interviews were conducted in either French or a local language by 15 experienced research assistants who had previously worked at CRSN and were recruited from within the study area. The training was conducted over a 1-week period through didactic and interactive sessions, including internal practice and problem-solving and piloting within Nouna town.

The study collected information on sociodemographics, behaviors, health practices, and health outcomes using tablet computers. Interviews were usually conducted outdoors near to participants' homes and were only carried out if the interview could not be overheard. Interviewers were assigned to potential participants at random without matching on any characteristics.

### Randomized controlled trial

We nested an individually randomized controlled trial of NVRCs within the ARISE Burkina Faso survey. All respondents provided verbal responses to nonsensitive questions in ARISE Burkina Faso. For questions relating to sexual experiences, violence, and mental health, all participants were randomized within the data collection application using a random number generator, with 50% probability to either the verbal method or the NVRC method. The randomization was conducted at the start of the NVRC module but ran in the background of the application, so the interviewer had no control over the response method used or which type of questions they had to pose.

### Nonverbal response card

**Figure 1:**
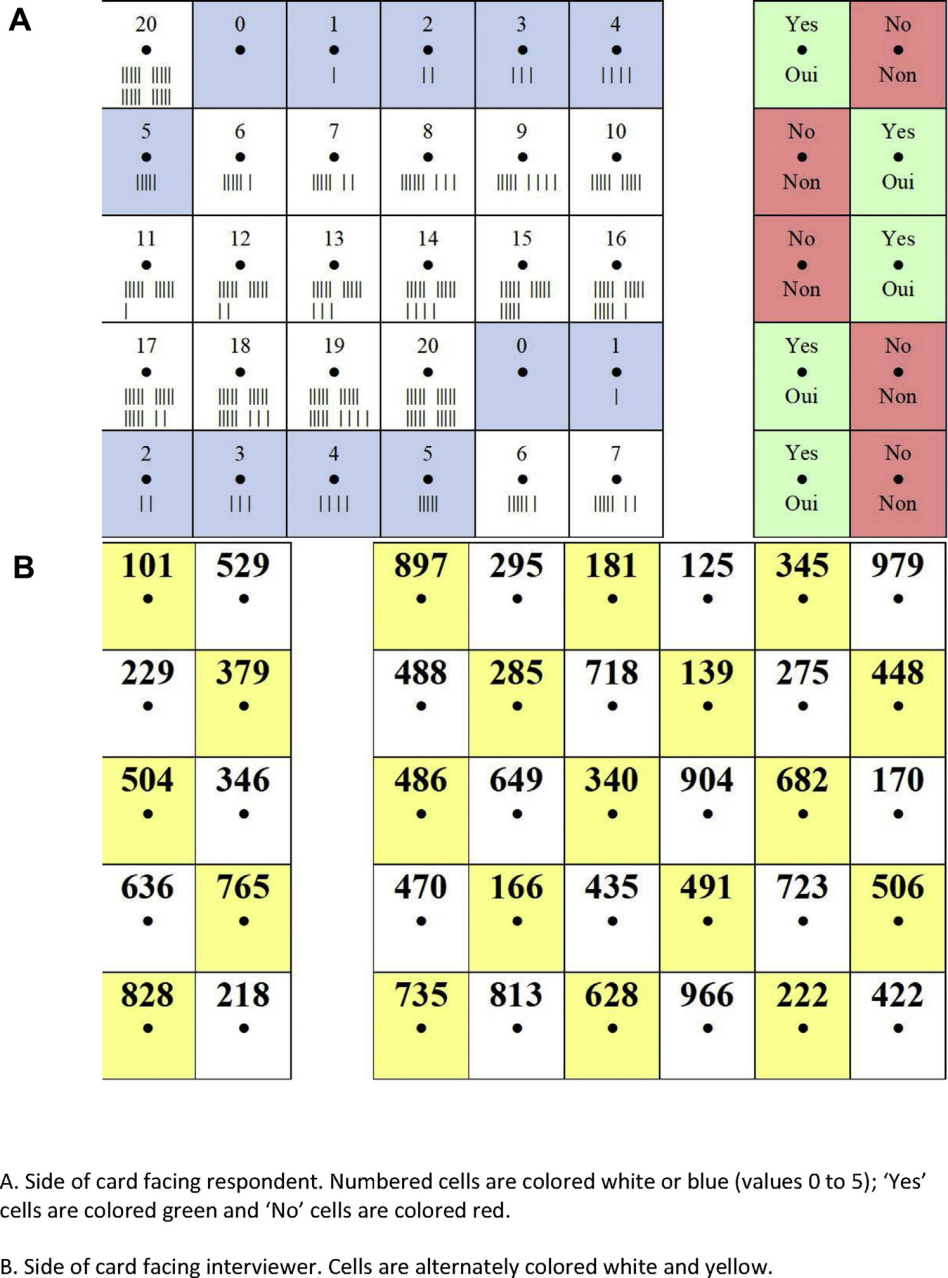
Nonverbal response card.

The NVRC is a two-sided, laminated card. Each side of the card is divided into 42 cells with a small hole punched through the center of each cell (Figure 1). Cells on the respondent side of the card contain written and color-coded responses. Nonnumeric responses are written in French and are color coded—green for “Yes” and red for “No.” Numeric responses range from 0 to 20 (e.g., for number of sexual partners and age at sexual initiation) and include hash marks for those unable to read Arabic numerals. Cells colored blue were also used for scale responses, for example, varying from 0 for “never” to 5 for “always.” The corresponding cells on the interviewer side of the card each contain a unique three-digit number.

Respondents hold the card so that they can see their side of the card and indicate their response to each question by inserting a stick through the hole in the relevant cell. The interviewer records only the three-digit number they see in the cell through which the stick is protruding. To minimize the risk or appearance of risk that the interviewer might recognize a response based on the position of the response cell, multiple cells containing the same value are provided on each card and the respondent is offered their choice of four cards with permuted response positions. Respondents are free to change the card they are using at any point in the interview. The card is divided into two panels, one with “Yes/No” responses and one with the numeric responses. If the respondent is distracted or not concentrating on the questions, the division of the card into the two panels potentially alerts the interviewer in the event that the respondent is providing a “Yes/No” response to a numeric response question or vice versa. After data collection is complete, the three-digit numeric codes are recoded to their corresponding response by a data analyst.

At the start of the sensitive question section, the interviewer demonstrates to the respondent how the cards work and uses a short set of practice questions to ensure that the respondent understands the procedure. The interviewer also holds his/her own small demonstration card through the sensitive questions section to remind the respondent how the card works. In this study, interview conditions were identical for both those using or not using the NVRC because interviewers and respondents did not know which arm they had been randomized to until they reached the NVRC module. At the start of the NVRC module, participants were explicitly told not to say your response so that the interviewer would never know the answer given.

### Measurement of key variables

The NVRC module included 15 “Yes/No” questions based on the Life Events Checklist in the Post-Traumatic Stress Disorder (PTSD) Checklist for DSM-5 [22,23] in the format “in all your life, have you ever experienced…,” including (1) physical assault; (2) sexual assault; and (3) other unwanted or uncomfortable sexual experiences. The module also asked respondents if they had ever had sexual intercourse and four questions of increasing sensitivity about sexual assault in the form “Have any of the following ever happened to you”: (1) “Someone made verbal jokes about wanting to have sex with you”; (2) “Someone touched you on your genital or breast when you did not want to be touched”; (3) “Someone forced you to have sex against your will but you escaped”; and (4) “Someone forced you to have sex against your will.”

As part of general field activities, the study team reviewed data every week for the first 3 weeks of data collection and provided interviewer-specific feedback where interviewers were generating responses that were markedly different from those of their peers or in the case of the NVRCs generating impossible values (e.g., an answer of “7” to the question, “Have you ever had sex?”).

To assess respondents' general proneness to social desirability bias, we asked all respondents to complete the Balanced Inventory of Desirable Responding [24], as translated into French by Sabourin et al. [25] and shortened from 40 to 16 items by Hart et al. [26], during the standard face-to-face interview.

### Statistical analysis

After describing the data, we evaluated the reliability and quality of the NVRC survey responses. Interviewer error in the form of data entry errors (limited in this case by automated data entry checks on the tablet computers) and respondent reporting error can occur with both the NVRC and verbal response methods. The cards are also susceptible to interviewer error in recording the three-digit response code onto the tablet. We measured nonresponse and error rates by response method, testing for significant differences using *χ*^*2*^ tests. We also evaluated how these rates changed over the 9-week survey period. As an additional check on the relative error rates of the two response methods, we looked at the number of married respondents who reported never having had sex, which we expect to be zero.

We tested the internal reliability of the card method by estimating Cronbach's alpha for the four measures of sexual violence (sexual assault, other unwanted sexual experience, attempted rape, and rape) and stratified by response method. These four measures can be conceptualized as capturing an underlying construct of experiencing sexual violence, with the expectation of strong positive correlation among the measures. We expected reported experience of sexual violence to be higher on all four items and with greater internal reliability among respondents who used the NVRC method compared with the verbal response method. For all alpha values, we calculated 95% confidence intervals (CIs) based on 1,000 bootstrap replications.

Finally, we evaluated interrater reliability under the assumption that interviewers were randomly assigned to participants; thus, we would expect to see similar prevalence rates for each outcome across interviewers within each arm. We therefore ran hierarchical linear regression models (respondents nested within interviewers) for each of the nine outcomes (one per study arm), containing only random intercepts for interviewers, to obtain intraclass correlation coefficients. Small and comparably sized intraclass correlation coefficients for the two response methods are evidence of consistent application of the two methods across respondents by the interviewers.

After evaluating reliability, we compared the level of affirmative responses across arms for each of the eight questions in the NVRC section, testing for significant differences using *χ*^*2*^ tests. We then conducted multivariable hierarchical Poisson regression analysis with robust error variance for the key outcomes of attempted forced sex and forced sex. We first evaluated whether any associations between NVRC use and affirmative responses were because of confounding by age, gender, marital status, potential PTSD status (based on the four-question Primary Care PTSD screen on which responding affirmatively to three or four items is considered indicative of probable PTSD [27]) or proneness to social desirability bias. We did not expect to see confounding here because the NVRC arm was randomly assigned. We then considered whether any of these variables modified the effect of the card on affirmative responses, comparing the Akaike Information Criterion for models without and with interaction terms for the relevant covariates before finally running models containing all the above covariates stratified by study arm for these two outcomes.

Approval for the broader ARISE study was obtained from the Harvard T.H. Chan School of Public Health Institutional Review Board. Approval for this study was obtained from the Institutional Ethics Committee of the CRSN; secondary data analysis was approved by the University College London's Research Ethics Committee. Oral assent was obtained from elders in each village. Written informed consent was obtained from each participant or from parents/guardians for respondents aged <18 years, with the minor's written assent. In cases of illiteracy, a literate witness assisted.

## Results

Of the 2,544 sampled adolescents, 1,644 were found and consented to participate. The great majority of those who did not participate had either moved out of the HDSS area (30.1%) or were not available for interview because of travel or work responsibilities (58.5%). In only 39 cases (4.4% of nonparticipants) did either the sampled individual or their parent decline consent. Among the 1,644 respondents, 785 (47.7%) were randomly assigned to the NVRC arm and 859 (52.3%) were randomly assigned to the verbal response method. No respondents refused to use the cards. Fifty-eight percent of respondents were male, and 42% were female (Table 1). Approximately half were in school at the time of interview, and only small proportions (largely female) were engaged or married. Religious and ethnic makeup reflected the local population. Consistent with the random assignment of the response methods, the two arms were largely indistinguishable with respect to gender, age, education, marital status, religion, and ethnicity.

**Table 1:** Descriptive statistics of ARISE Burkina Faso respondents

	All respondents	Verbal response	NVRC
N	1,644	859	785
Female			
12–15 years	25.0%	27.1%	22.8%
16–17 years	9.8%	10.7%	8.8%
≥18 years	7.5%	7.1%	7.9%
Male			
12–15 years	32.4%	29.3%	35.7%
16–17 years	13.8%	13.9%	13.8%
≥18 years	11.5%	11.9%	11.1%
Education			
Currently in school	50.0%	52.0%	47.8%
Marital status			
Engaged to be married	2.7%	2.8%	2.6%
Ever married	6.8%	6.9%	6.8%
Religion			
Muslim	69.2%	67.5%	70.1%
Catholic	20.7%	21.9%	19.4%
Protestant	6.9%	7.7%	6.1%
Animist	3.2%	2.9%	3.6%
Ethnicity			
Bwaba	19.9%	21.5%	18.1%
Dafin	38.1%	36.1%	40.3%
Mossi	17.6%	17.4%	17.8%
Peulh	10.1%	9.4%	10.8%
Samo	12.5%	13.4%	11.6%
Other	1.8%	2.2%	1.4%

ARISE = Africa Research, Implementation Science and Education; NVRC = nonverbal response card.

The combined item-specific nonresponse and error rates were very low for both NVRC and verbal response arms, ranging from .3% to 1.7% across questions (Table 2). Although the NVRC nonresponse and error rates were generally higher than those for the verbal response method, no differences were statistically significant. The nonresponse/error rate for the NVRC method also declined over the course of the survey: from 2.3% during the first interview week to .2% in the fourth to ninth weeks (results not shown). Only six married individuals reported never having had sex: four in the NVRC arm and two among verbal respondents; the difference was not statistically significant.

**Table 2:** Comparison of reported violence responses within the NVRC survey section by randomization arm

	Nonresponse/errors				Affirmative responses			
	Verbal	NVRC	*χ*^*2*^	*p* value	Verbal	NVRC	*χ*^*2*^	*p* value
Lifetime traumatic events								
Physical assault	.1%	.4%	1.2	.28	34.5%	27.0%	10.8	.001
Assault with a weapon	.1%	.5%	2.1	.15	4.8%	7.3%	4.6	.03
Sexual assault	.5%	.3%	.5	.48	2.6%	4.9%	6.0	.01
Other unwanted sexual experience	.3%	.3%	.1	.73	1.9%	5.5%	15.5	<.001
Ever had sexual intercourse	.7%	.4%	.8	.39	16.2%	15.2%	.3	.60
Someone joked about wanting to have sex	.1%	.6%	3.1	.08	12.1%	12.9%	.3	.61
Someone touched genitals/breasts	.9%	.9%	<.1	.93	17.5%	15.7%	1.0	.32
Someone tried to force sex but failed	.3%	.8%	1.3	.26	4.7%	7.6%	6.0	.01
Someone forced sex	.5%	1.0%	1.7	.19	2.9%	5.8%	8.2	.004

All *χ*^*2*^ tests have one degree of freedom.

NVRC = nonverbal response card.

Internal reliability for the four questions on sexual assault was moderately high and comparable for the two study arms. Cronbach's alpha was .68 (95% CI: .61–.75) for the card respondents and .70 (95% CI: .58–.82) for respondents in the verbal arm. These values suggest that those using the nonverbal cards gave answers no less reliable than those using verbal response. Interrater reliability for the questions regarding experience of physical violence, sex, and sexual violence varied across arm but not in a systematic manner (Table 3). The intraclass correlation coefficients were comparatively small for most questions and generally nonsignificantly smaller in the NVRC arm of the study compared with the verbal arm.

**Table 3:** Interviewer-level intraclass correlation coefficients with 95% confidence intervals

	Verbal	NVRC
Lifetime traumatic events		
Physical assault	.28 (.14, .48)	.19 (.08, .39)
Assault with a weapon	.03 (.01, .07)	.03 (.01, .06)
Sexual assault	.00 (.00, .00)	.04 (.02, .08)
Other unwanted sexual experience	.01 (.00, .03)	.05 (.02, .13)
Ever had sexual intercourse	.02 (.01, .05)	.01 (.00, .06)
Someone joked about wanting to have sex	.09 (.03, .20)	.03 (.01, .07)
Someone touched genitals/breasts	.17 (.08, .34)	.04 (.02, .10)
Someone tried to force sex but failed	.03 (.01, .06)	.03 (.01, .06)
Someone forced sex	.03 (.01, .11)	.01 (.00, .09)

Each result arises from a separate two-level regression model containing only random intercepts by interviewer.

NVRC = nonverbal response card.

**Figure 2:**
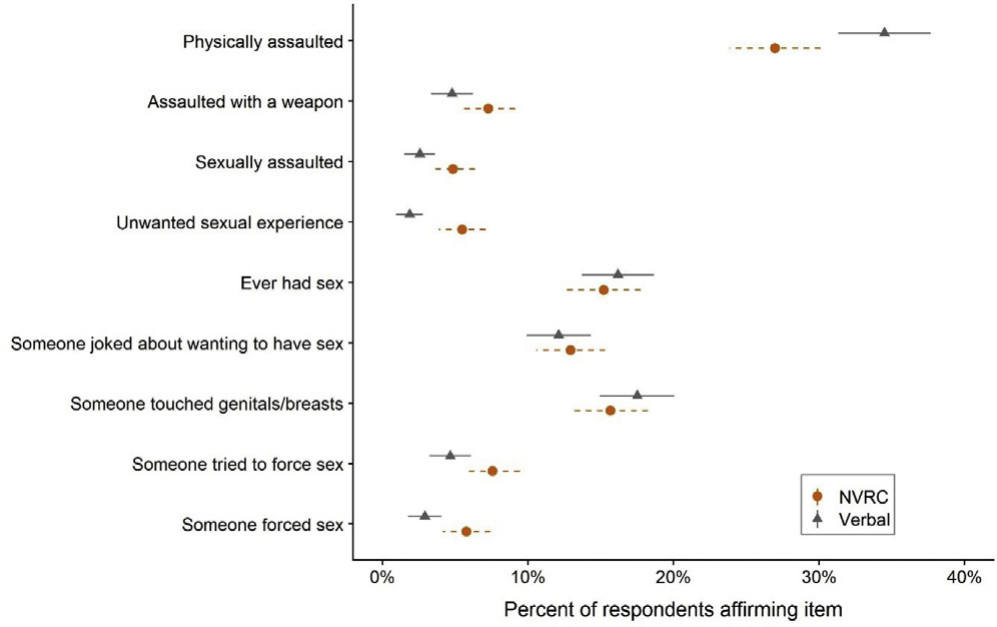
Affirmative response rates in NVRC section by randomization arm. NVRC = Nonverbal response card.

Verbal respondents were significantly more likely to report having ever been physically assaulted than respondents who used the cards but slightly less likely to report having been assaulted with a weapon (Figure 2 and Table 2). For the sexual experience questions, the rates of reporting ever having had sexual intercourse, joking about wanting to have sex, and sexual touching were not significantly different across arms. However, respondents using the NVRC method reported significantly higher rates of unwanted sexual experiences, sexual assault, attempted forced sex, and forced sex.

In bivariate associations, respondents in the NVRC arm reported 1.60 times the prevalence of attempted forced sex (95% CI: 1.04–2.45) and 1.88 times the prevalence of forced sex (95% CI: 1.08–3.28) as the verbal response arm. This relationship was not significantly affected by adjustment for covariates (Table 4, Model 1). In adjusted regression models, forced sex rose with age and was more common among females compared with males; the pattern was less clear for attempted forced sex. Engagement and marriage, although rare in this sample, were associated with greater reports of both outcomes, as was screening positive for PTSD. Scoring highly on proneness to social desirability was associated with less reporting of forced sex, which might be expected if such experiences were stigmatized.

**Table 4:** Prevalence ratios with 95% confidence intervals for reported lifetime history of unwanted forced sex

	Attempted forced sex			Forced sex		
	Model 1	Model 2	Model 3	Model 1	Model 2	Model 3
NVRC versus standard	1.65 (1.03, 2.64)	1.63 (1.02, 2.59)	1.37 (.86, 2.18)	1.98 (1.19, 3.32)	1.93 (1.12, 3.32)	1.68 (1.05, 2.68)
Female, 12–15 years	1.00	1.00	1.00	1.00	1.00	1.00
Female, 16 or 17 years	1.40 (.77, 2.54)	1.40 (.75, 2.63)	1.30 (.74, 2.28)	1.14 (.50, 2.59)	1.16 (.52, 2.61)	1.11 (.52, 2.37)
Female, 18–20 years	.89 (.57, 1.40)	.87 (.56, 1.34)	1.01 (.58, 1.76)	2.39 (1.29, 4.42)	2.35 (1.31, 4.19)	2.30 (1.44, 3.67)
Male, 12–15 years	.44 (.15, 1.34)	.44 (.14, 1.34)	.50 (.16, 1.56)	.28 (.09, .86)	.27 (.09, .78)	.33 (.10, 1.09)
Male, 16 or 17 years	.55 (.20, 1.50)	.54 (.20, 1.43)	.58 (.21, 1.59)	.43 (.19, .97)	.44 (.20, .95)	.46 (.20, 1.09)
Male, 18–20 years	1.02 (.47, 2.22)	1.05 (.49, 2.23)	1.03 (.48, 2.23)	.89 (.44, 1.79)	.93 (.47, 1.83)	.88 (.42, 1.86)
Single	1.00	1.00	1.00	1.00	1.00	1.00
Engaged	3.41 (1.62, 7.16)	3.33 (1.65, 6.74)	3.31 (1.82, 6.02)	1.53 (.49, 4.80)	1.39 (.41, 4.74)	1.84 (.61, 5.59)
Married	2.11 (1.14, 3.90)	2.19 (1.19, 4.02)	1.84 (1.05, 3.23)	1.40 (.69, 2.87)	1.44 (.70, 2.94)	1.32 (.65, 2.67)
PC-PTSD screen positive			3.55 (2.20, 5.71)			4.25 (2.03, 8.91)
Social desirability level						
Lowest quintile		1.00			1.00	
Second quintile		.81 (.52, 1.26)			.72 (.35, 1.47)	
Middle quintile		.64 (.40, 1.03)			.65 (.29, 1.45)	
Second highest quintile		.41 (.21, .81)			.38 (.10, 1.42)	
Highest quintile		.67 (.40, 1.10)			.31 (.13, .72)	
N	1,633	1,633	1,619	1,630	1,630	1,617
AIC	800.2	801.1	766.2	590.5	588.9	563.6
AIC for interaction with^a^:						
Age, gender, marital status	800.7			593.6		
BIDR quintiles		800.6			591.5	
PC-PTSD screener			765.8			565.4

All results are from hierarchical (respondents nested in interviewers) Poisson regression models.

AIC = Akaike Information Criterion; BIDR = Balanced Inventory of Desirable Responding; NVRC = nonverbal response cards; PC-PTSD = Primary Care Post-Traumatic Stress Disorder screening tool.

a AIC values below here are from models in which the named variables were interacted with NVRC arm.

Adding interaction terms for each of the covariates did not improve the Akaike Information Criterion values for either model (Table 4), suggesting an absence of a response method effect by proneness to social desirability. This result suggests that the nonverbal cards are equally effective among respondents at different levels of proneness to social desirability bias, with the proviso that the Balanced Inventory of Desirable Responding has not been validated in this study population.

## Discussion

In this randomized trial among adolescents in a poor, rural part of Burkina Faso, we found that NVRCs—which protect respondent privacy and confidentiality—led to more reports of the least common and most sensitive experiences we asked about. Prevalence levels were not significantly different by response method for the more common experiences of physical violence, having had someone joke about having sex with the respondent, and unwanted sexual touching. However, for less common experiences, notably sexual assault, having someone try but fail to force sex and having someone force sex, NVRC arm respondents were up to twice as likely to answer affirmatively. When we examined the forced sex questions in detail, we did not find evidence that the effect of NVRCs was modified by other predictors.

NVRC responses were no more prone to item-specific nonresponse than verbal response, and the level of internal reliability was higher. The already-low error rate associated with the cards fell as the survey progressed. Informal feedback from the interviewers suggested that most adolescent respondents quickly understood the card method and experienced no difficulty in using the cards. As well as producing low item-specific error rates, the NVRCs did not lead to less coherent sets of responses. Respondents in the NVRC arm tended to provide groups of sexual violence question responses that were just as internally consistent, as indicated by values of Cronbach's alpha, as respondents in the normal, verbal response arm. This result suggests that respondents using the verbal method reported lower levels of all four types of sexual violence in a consistent manner, and respondents using the cards tended to report higher levels of sexual violence in a consistent manner.

Our work extends previous use of the NVRC method in Ethiopia to a different population. We find that the NVRC method offers a low-cost, robust method for reducing social desirability bias in a population with high rates of illiteracy. It is notable that our study results for sexual violence align with results from a randomized trial test of the NVRC method in a sample of youth in Ethiopia, which found that the reported prevalence of rape was twice as high among respondents who used the NVRC method compared with the verbal response method [16]. These comparable findings of differential reporting of coerced sexual experience in two very different settings provide external validation for the NVRC method and evidence of the likely underreporting of sexual violence in adolescent health surveys that use the verbal response method.

Important future avenues for investigation with NVRCs include testing how differences in reporting change on repeated use by the same respondents, evaluating whether it is the nonverbal or confidentiality aspects of the cards that lead to greater reporting of more socially unacceptable responses, and extending the use of these cards to older adults who may also face literacy and social desirability biases and may or may not be able to understand the novel methodology as quickly as did the adolescents in our study. Using qualitative interviewing of respondents and interviewers will be particularly important in evaluating feasibility and acceptability and finding and fixing any barriers to effective use.

A central limitation of NVRCs is that interviewers have less control over the interview process when using these cards than they usually would: they are unable to identify illogical responses, such as numerical responses that are clearly out of range (although this was a relatively rare occurrence in this study). Ultimately, NVRCs represent a midpoint between an interviewer-run interview where privacy cannot be assured and a respondent-run interview where interviewers' ability to use their expertise to help participants understand and respond validly is limited.

This study also has limitations, notably that there is no gold standard against which to test the violence-related responses. Although we found that the responses given using NVRCs were at least as coherent as verbal responses and similarly associated with key predictors of violence, further comparison using a verifiable response would be ideal. It is also unclear how broadly our NVRC findings can be generalized: in terms of geography beyond sub-Saharan Africa, in terms of literacy status beyond largely illiterate populations, or in terms of age beyond adolescents. These are testable in future work, however, which would help validate our finding and allow triangulation with this study.

## Conclusion

We have demonstrated that NVRCs are practical and beneficial way of reducing socially desirable misreporting on sensitive topics in adolescents with low education and literacy attainment. These cards protect privacy during the interview process and are likely a good choice for sensitive topics where interviewer guidance can improve comprehension and willingness to respond.
